# Front‐line treatment of ceritinib improves efficacy over crizotinib for Asian patients with anaplastic lymphoma kinase fusion NSCLC: The role of systemic progression control

**DOI:** 10.1111/1759-7714.13221

**Published:** 2019-10-15

**Authors:** Shih‐Hao Huang, Allen Chung‐Cheng Huang, Chin‐Chou Wang, Wen‐Chen Chang, Chien‐Ying Liu, Stelios Pavlidis, Ho‐Wen Ko, Fu‐Tsai Chung, Ping‐Chih Hsu, Yi‐Ke Guo, Chih‐Hsi Scott Kuo, Cheng‐Ta Yang

**Affiliations:** ^1^ Division of Thoracic Oncology, Department of Thoracic Medicine Chang Gung Memorial Hospital, Chang Gung University, College of Medicine Taipei Taiwan; ^2^ Division of Pulmonary & Critical Care Medicine Kaohsiung Chang Gung Memorial Hospital Kaohsiung Taiwan; ^3^ Department of Medical Oncology Chang Gung Memorial Hospital, Chang Gung University Taipei Taiwan; ^4^ Data Science Institute, Department of Computing Imperial College London London UK

**Keywords:** ALK, ceritinib, crizotinib, NSCLC

## Abstract

**Background:**

Approximately 3%–5% of lung adenocarcinoma is driven by anaplastic lymphoma kinase (ALK) fusion oncogene, whose activity can be suppressed by multiple ALK inhibitors. Crizotinib and ceritinib have demonstrated superior efficacy to platinum‐based chemotherapy as front‐line treatment for patients with ALK‐positive advanced non‐small cell lung cancer (NSCLC). However, the direct comparison between them in the front‐line setting remains lacking.

**Methods:**

A total of 48 patients with ALK‐positive, previously untreated advanced NSCLC, who received crizotinib and ceritinib as front‐line treatment were retrospectively investigated. The efficacy and pattern of disease progression were analyzed.

**Results:**

Patients receiving ceritinib treatment were significantly younger than those receiving crizotinib treatment (52.0 vs. 63.0, *P* = 0.016). The median progression‐free survival (PFS) was significantly longer with ceritinib than with crizotinib treatment (32.3 vs. 12.9 months; log‐rank *P* = 0.020); the hazard ratio for disease progression or death, 0.27 (95% CI, 0.08–0.90; *P* = 0.033). An objective response was noted in all patients in the ceritinib group and in 23 patients in the crizotinib group (74.2%; 95% CI, 59.0 to 88.5). The rate of systemic progression was significantly lower over time with ceritinib treatment compared to crizotinib treatment (cause‐specific hazard ratio, 0.21; 95% CI 0.06–0.73; *P* = 0.014). Serious adverse events were noted in one (2.9%) patient showing elevated liver function in the crizotinib group and three (23.1%) patients showing diarrhea in the ceritinib group. Dose reduction was needed in five out of 13 (38.5%) patients receiving ceritinib treatment.

**Conclusion:**

Ceritinib showed higher efficacy associated with a better control of systemic progression compared to crizotinib for the front‐line treatment of ALK‐positive advanced NSCLCs.

## Introduction

Advanced non‐small cell lung cancer (NSCLC) is a deadly disease with a dismal prognosis.^1^ However, a treatment strategy targeting the inhibition of driving mutations has revolutionized the survival in some patients.^2^ One specific patient group which has experienced a dramatic improvement in survival are those patients with anaplastic lymphoma kinase (ALK) fusion NSCLC who receive treatment with ALK tyrosine kinase inhibitors (ALKi).

Crizotinib, the first FDA‐approved ALKi for the treatment of advanced ALK‐positive NSCLC, has shown superior efficacy compared to the standard of care chemotherapy docetaxel and pemetrexed‐cisplatin doublet in the second‐ and first‐line setting, respectively.^3^, ^4^ Disease progression usually occurs around 10 to 12 months during crizotinib treatment where the pattern of progression involves a high frequency of brain metastasis which targets the brain as a sanctuary site.^5^, ^6^ This finding can be partly attributed to the drug transporters located at the blood brain barrier that actively efflux the therapeutic drugs and thereby reduce their concentrations in the brain tissue.^7^ A previous study in a mouse model indicated that gene knockout mice lacking the drug transporters P‐glycoprotein (ABCB1) and breast cancer resistance protein (ABCG2) had a 20‐ to 70‐fold higher brain accumulation of crizotinib than their wild‐type counterparts.^8^ In light of this, the second generation ALKi alectinib, which is not a substrate of the drug transporters ABCB1 and ABCG2, has shown a superior efficacy over crizotinib in comparative studies.^9^, ^10^ This advantage over crizotinib was mainly attributed to a lower incidence of brain progression during the treatment of alectinib, whereas the incidence of systemic progression between the two drugs was similar.^10^

Apart from alectinib which features a promising brain tissue penetration, another second generation ALKi ceritinib is characterized by a relatively high potency against ALK tyrosine kinase compared to crizotinib and alectinib.^11^, ^12^ Ceritinib was the first FDA‐approved second generation ALKi for the treatment of ALK‐positive NSCLC. It has shown promising efficacy over chemotherapy for patients with disease progression from crizotinib treatment and also patients who have not been previously treated.^13^, ^14^ Given the high potency of ceritinib, both against wild‐type ALK as well as a number of gatekeeper mutations,^15^ ALK‐positive NSCLC patients treated with ceritinib may have a better control of systemic progression than those treated with crizotinib, albeit evidence of the direct comparison between the two drugs is scarce.

On the other hand, the adequate penetration of ceritinib to brain tissues for the control and prevention of brain metastasis remains a controversial issue. A previous study of a mouse model had shown that the penetration of ceritinib, as measured by the ratio of plasma to cerebral spinal fluid concentration, was only around 15% in which the active efflux of ceritinib by the drug transporters ABCB1 and ABCG2 still accounted for its reduced penetration to brain tissues.^16^ This finding may also partly be associated with a diverse intracranial efficacy of ceritinib, as the intracranial objective response rate of brain metastasis ranged between 40% to 70% for ALKi‐naïve patients who received ceritinib treatment.^14^, ^17^, ^18^

In the meantime, no dedicated clinical trial which directly compares the therapeutic outcome between ceritinib and crizotinib is available, except a few reports which indirectly compare the two drugs using the patients that propensity score‐matched from individual clinical trials originally conducted for the study of crizotinib and ceritinib, respectively.^19^, ^20^ These indirect comparisons are insightful for the analysis of the primary outcomes consistently defined across the original trials, such as progression‐free or overall survival, although they might not be feasible for the analysis of the outcomes, such as the pattern of disease progression, as these were not consistently defined across the trials. In this study, we retrospectively compared a group of patients who received either crizotinib or ceritinib as the front‐line treatment and the treatment efficacy, pattern of disease progression and toxicity profile were analyzed.

## Methods

### Patients

Between January 2015 and August 2018, a total of 110 patients with advanced or metastatic ALK fusion NSCLCs diagnosed by Ventana ALK (D5F3) CDx immunohistochemistry assay (Roche Diagnostics, USA) at Chang Gung Memorial Hospital were retrospectively reviewed using Chang Gung Research Database. A total of 48 patients had ALK inhibitors as the front‐line treatment, in which 35 patients received crizotinib 250 mg twice daily and 13 patients received ceritinib 750 mg or 450 mg once daily. Progression‐free survival (PFS) was defined as the interval between the date of the start of ALK inhibitors and the date of either radiologically‐documented progression or death. The treatment response, defined as complete response (CR), partial response (PR), stable disease (SD), and progressive disease (PD), was evaluated according to the Response Evaluation Criteria in Solid Tumors (RECIST) version 1.1. The pattern of post‐ALK inhibitor disease progression was also reviewed and defined as either systemic progression without prior CNS progression/death or CNS progression without prior systemic progression/death. The toxicities noted during ALK inhibitor treatment were systemically reviewed and the toxicity was graded according to the National Cancer Institute Common Toxicity Criteria, version 5.0. The study was approved by the Ethics Committee of Chang Gung Memorial Hospital.

### Statistical analysis

The Mann‐Whitney test was used to determine the statistical significance between two groups of continuous variables; Fisher's exact tests were used for categorical variables. The Kaplan‐Meier survival curve was analyzed using the R package survival, and the hazard ratio was analyzed using the Cox regression model. For the propensity score matching analysis, the ceritinib versus the crizotinib group served as the dependent variable; the covariates used included age, smoking history, Eastern Cooperative Oncology Group performance status (ECOG PS) and brain metastasis. The coefficient for each covariate was determined by logistic regression analysis, and the propensity score of each individual was calculated as the sum of the product of each coefficient and the value of each covariate. The pairs of ceritinib and crizotinib individuals with equivalent propensity scores were selected in a 1:2 manner using the R package MatchIt. The patterns of post‐ALK inhibitor disease progression were treated as competing risk events of which the cumulative incidence functions were calculated.^21^ The modified Cox regression model for the subdistribution hazard of the cumulative incidence function was applied^22^ to calculate the hazard of disease progression from a given pattern in the presence of competing event using the R package cmprsk. All the reported *P*‐values were two‐sided, and a *P*‐value less than 0.05 was considered to be statistically significant. All the data were analyzed using SPSS 10.1 (SPSS Corp., Chicago, IL, USA).

## Results

### Baseline patient characteristics

Clinical data from 48 patients of ALK fusion advanced or metastatic NSCLC receiving firstline ALK inhibitor treatment (35 in the crizotinib group and 13 in the ceritinib group) were extracted from the Chang Gung Research Database. Most of the baseline clinical characteristics, including sex, ECOG PS, brain metastasis and treatment for brain metastasis, were well balanced between the crizotinib and ceritinib groups (Table 1); the age of the patients receiving crizotinib was significantly older than the patients receiving ceritinib (63 [56–69] vs. 54 [37–58]; *P* = 0.016) and the frequency of smoking history in crizotinib group was lower than the ceritinib group (22.9% vs. 38.5%; *P* = 0.298; Table 1). The median duration of follow‐up was 9.6 months for the crizotinib group and 36.0 months for the ceritinib group. At the time of analysis, 11 patients (31.4%) had discontinued treatment in the crizotinib group and five patients (38.5%) had discontinued treatment in the ceritinib group.

**Table 1:** Baseline characteristics of the study population

Variables, *n* (%)	Total (*n* = 48)	Crizotinib (*n* = 35)	Ceritinib (*n* = 13)	*P*‐value
Age, median (range), year	60 (50–67)	63 (56–69)	54 (37–58)	0.016
Sex				
Male	24 (50.0)	17 (48.6)	7 (53.8)	1.000
Female	24 (50.0)	18 (51.4)	6 (46.2)	
Smoking history				
Smoker/ex‐smoker	13 (27.1)	8 (22.9)	5 (38.5)	0.298
Nonsmoker	35 (82.9)	27 (77.1)	8 (61.5)	
ECOG PS				
0 or 1	46 (95.8)	33 (94.3)	13 (100.0)	1.000
2	2 (4.2)	2 (5.7)	0	
Staging				
IIIC	3 (6.3)	3 (8.6)	0	0.553
IV	45 (93.7)	32 (91.4)	13 (100.0)	
Histology				
Adenocarcinoma	48 (100.0)	35 (100.0)	13 (100.0)	1.000
Brain metastasis				
Yes	16 (33.3)	12 (34.3)	4 (30.8)	1.000
No	32 (66.7)	23 (65.7)	9 (69.2)	
Treatment for brain metastasis (No./total No.)				
WBRT	7/8 (87.5)	5/6 (83.3)	2/2 (100)	1.000
SRS	1/8 (12.5)	1/6 (16.7)	0	

ECOG PS, Eastern Cooperative Oncology Group performance status; SRS, stereotactic radiosurgery; WBRT, whole‐brain radiotherapy.

### Treatment efficacy between crizotinib and ceritinib

**Figure 1:**
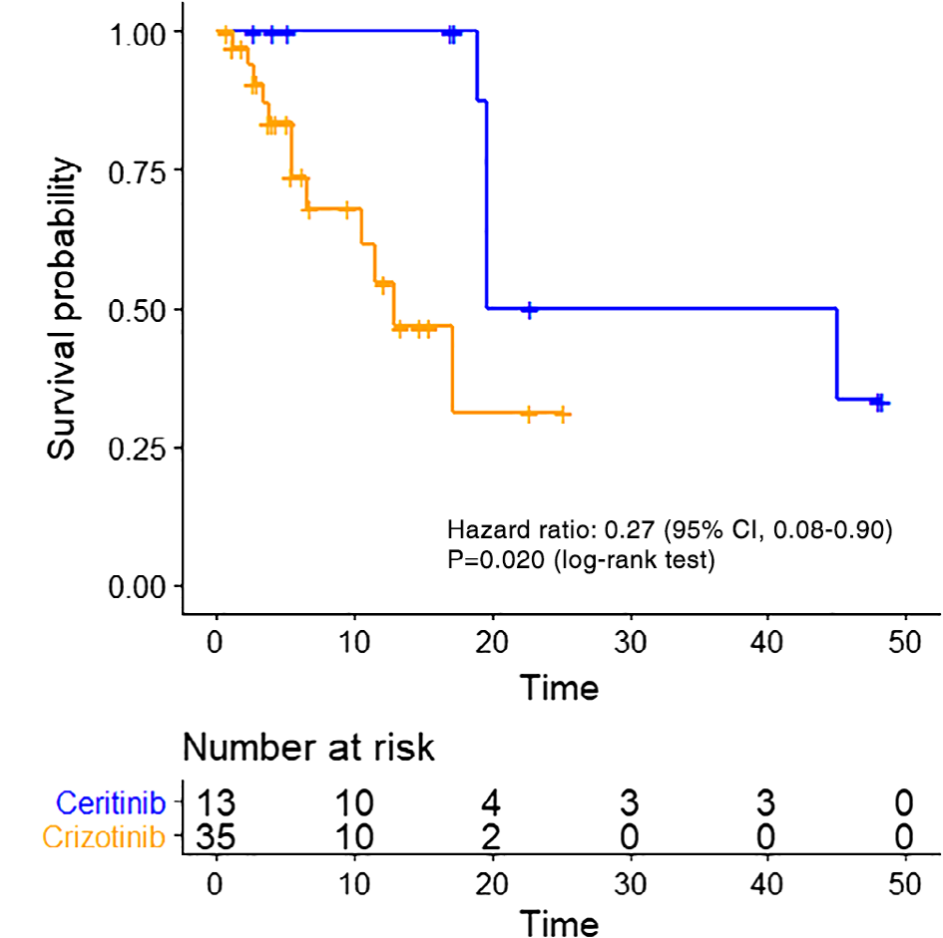
Kaplan‐Meier curve of PFS between crizotinib and ceritinib in the study population. ALK inhibitor (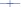) ceritinib and (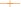) crizotinib.

**Figure 2:**
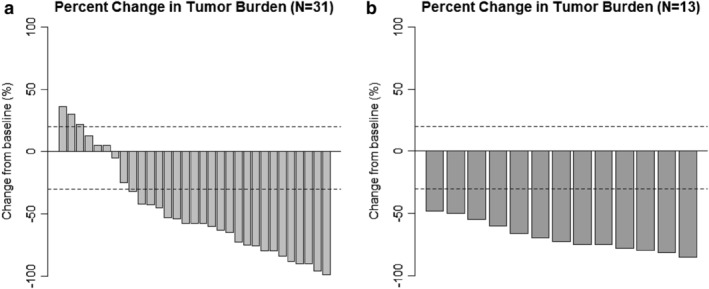
Waterfall plot for the percentage decrease of tumor burden of each patient with evaluable tumor response in (**a**) crizotinib group, (**b**) ceritinib group.

At the time of data cutoff, a total of 17 events of disease progression or death had been noted (12 of 35 patients [34.3%] in the crizotinib group and five of 13 patients [38.5%] in the ceritinib group). The 18‐month PFS rate was significantly higher in the ceritinib group than in the crizotinib group (87.5% [95% confidence interval,^23^ 65.5 to 100.0] vs. 31.1% [95% CI, 15.8 to 46.4]); the hazard ratio for disease progression or death was 0.27 (95% CI, 0.08–0.90; *P* = 0.033); and the median PFS with ceritinib treatment was 32.3 months (95% CI, 19.6 to not estimable), as compared with 12.9 months (95% CI, 10.6 to not estimable; log‐rank test *P* = 0.020; Fig 1) with crizotinib treatment. The tumor burden was estimable for an objective response in 44 patients (31 with crizotinib and 13 with ceritinib), in which the CR, PR, SD and PD were 3.2%, 71.0%, 16.1% and 9.7% for the crizotinib group and 100% of PR in the ceritinib group (Fig 2); a better response rate was noted for the patients receiving ceritinib treatment (100.0% vs. 74.2%, *P* = 0.082; Table 2).

**Table 2:** Objective response rate in the study population

Variables, *n* (%)	Crizotinib (*n* = 31)	Ceritinib (*n* = 13)
Response		
No. of patients	23	13
% (95% CI)	74.2 (61.3–87.1)	100 (100–100)*
Complete response, No. (%)	1 (3.2)	0
Partial response, No. (%)	22 (71.0)	13 (100.0)
Stable disease, No. (%)	5 (16.1)	0
Progression disease, No. (%)	3 (9.7)	0

* *P* = 0.082 for the comparison between ceritinib and crizotinib.

### Propensity score‐matched cohort analysis

**Figure 3:**
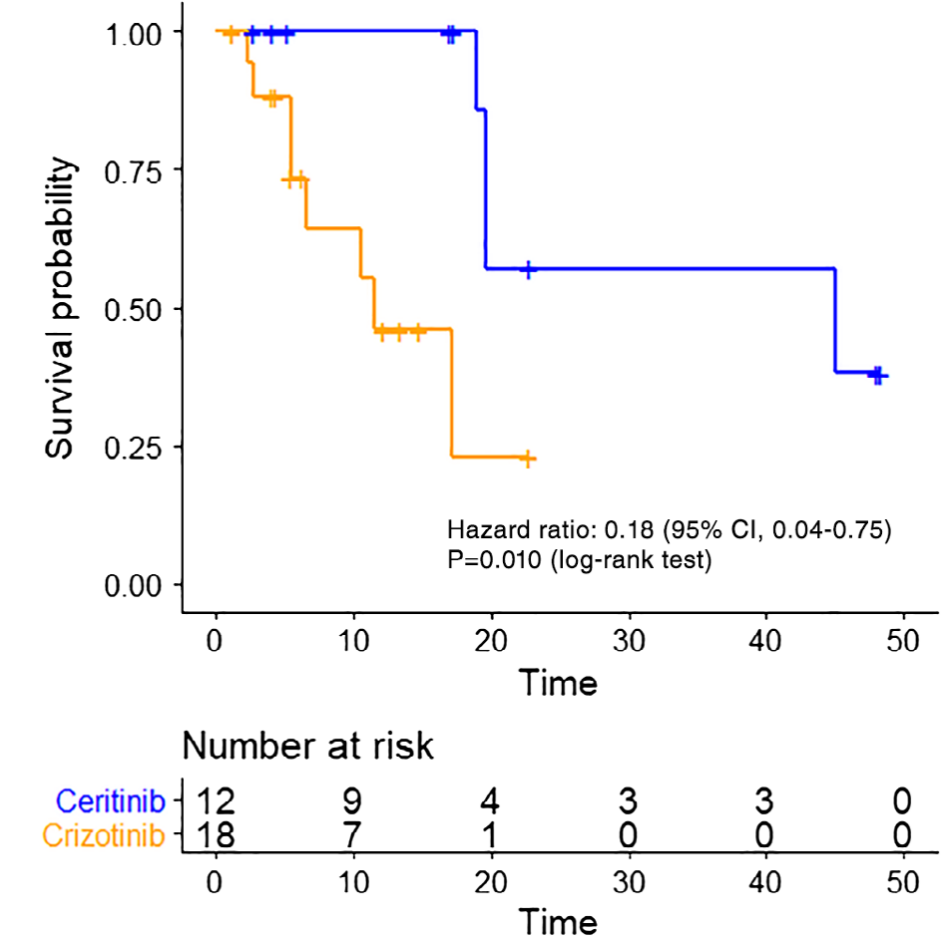
Kaplan‐Meier curve of PFS between crizotinib and ceritinib in the propensity score‐matched cohort. ALK inhibitor (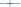) ceritinib and (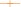) crizotinib.

To address the confounding factors that can potentially affect the therapeutic efficacy between the two groups (ie, mainly the age and smoking history), we performed propensity score matching analysis. After 1:2 matching according to the individual's propensity score—one who belonged to the ceritinib group and two who belonged to the crizotinib group—patients with balanced clinical profiles were selected (Table 3). Among the propensity score‐matched subpopulation, the median PFS of the ceritinib group remained longer than that of patients in the crizotinib group (45.0 months [95% CI, 19.6 to not estimable] vs. 11.5 months [95% CI, 6.6 to not estimable]; log‐rank test *P* = 0.010; Fig 3); the 18‐months PFS rate (85.7% [95% CI, 68.8 to 100.0] vs. 23.0% [95% CI, 3.6 to 42.4]) and the hazard ratio for disease progression or death, 0.18 (95% CI, 0.04–0.75; *P* = 0.018; Fig 3) were also significantly better for patients receiving ceritinib treatment.

**Table 3:** Characteristics of the propensity score‐matched cohort

Variables, *n* (%)	Total (*n* = 30)	Crizotinib (*n* = 18)	Ceritinib (*n* = 12)	*P*‐value
Age, median (range), year	55 (45–62)	56 (47–65)	54 (42–59)	0.472
Sex				
Male	14 (46.7)	8 (44.4)	6 (50.0)	1.000
Female	16 (53.3)	10 (55.6)	6 (50.0)	
Smoking history				
Smoker/ex‐smoker	11 (36.7)	6 (33.3)	5 (41.7)	0.712
Nonsmoker	19 (63.3)	12 (66.7)	7 (58.3)	
ECOG PS				
0 or 1	29 (96.7)	17 (94.4)	12 (100.0)	1.000
2	1 (3.3)	1 (5.6)	0	
Staging				
IIIC	2 (6.7)	2 (11.1)	0	0.503
IV	28 (93.3)	16 (88.9)	12 (100.0)	
Histology				
Adenocarcinoma	30 (100.0)	18 (100.0)	12 (100.0)	1.000
Brain metastasis				
Yes	7 (23.3)	4 (22.2)	3 (25.0)	1.000
No	23 (76.7)	14 (77.8)	9 (75.0)	
WBRT for brain metastasis	4 (13.3)	2 (11.1)	2 (16.7)	1.000

ECOG PS, Eastern Cooperative Oncology Group performance status; WBRT, whole‐brain radiotherapy.

### Pattern of disease progression between crizotinib and ceritinib

**Figure 4:**
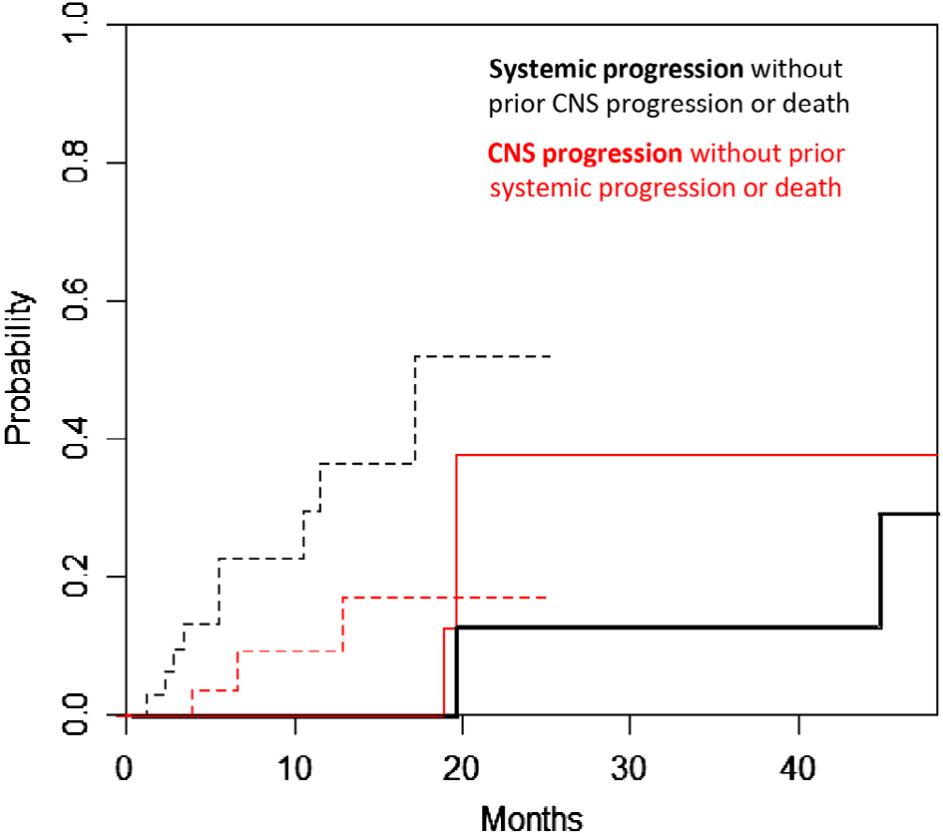
Cumulative incidence of systemic progression (color code black) and CNS progression (color code red) between the crizotinib (broken line) and ceritinib (solid line) groups. (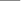) Ceritinib, (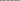) crizotinib, (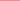) ceritinib and (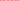) crizotinib.

The pattern of post‐treatment disease progression, either systemic or CNS progression, was analyzed in a fashion of competing risk based on a cumulative incidence rate. The rate of systemic progression was significantly lower over time with ceritinib treatment as compared to crizotinib treatment (cause‐specific hazard ratio, 0.21; 95% CI 0.06–0.73; *P* = 0.014, Fig 4), where 2 (15.4%) patients in the ceritinib group and nine (25.7%) patients in the crizotinib group had an event of systemic progression. In addition, the rate of CNS progression was equivalent between ceritinib and crizotinib treatment (cause‐specific hazard ratio, 1.27; 95% CI 0.33–4.94; *P* = 0.730, Fig 4).

### Adverse events profile

The most commonly noted all grade adverse events in patients in the crizotinib group included nausea/poor appetite (seven patients; 20.0%), diarrhea (seven patients; 20.0%) and elevated AST/ALT (12 patients; 34.3%). In the ceritinib group, the most commonly noted all grade adverse events included diarrhea (seven patients; 53.8%), nausea/poor appetite (six patients; 46.2%) and vomiting (three patients; 23.1%; Table 4). Serious adverse events of grade 3–5 were noted in one (2.9%) patient showing elevated AST/ALT in the crizotinib group and three (23.1%) patients showing diarrhea in the ceritinib group. Dose reduction was needed in five out of 13 (38.5%) patients receiving ceritinib treatment.

**Table 4:** Treatment‐related adverse events

	Crizotinib (*n* = 35)		Ceritinib (*n* = 13)	
Frequency *n* (%)	Any grade	Grade 3–5	Any grade	Grade 3–5
Nausea/poor appetite	7 (20.0)	1 (2.9)	6 (46.2)	2 (15.4)
Diarrhea	7 (20.0)	1 (2.9)	7 (53.8)	3 (23.1)
Vomiting	4 (11.4)	1 (2.9)	3 (23.1)	1 (7.7)
Elevation of AST/ALT	8 (22.9)	1 (2.9)	5 (38.5)	1 (7.7)
Peripheral edema	5 (14.3)	0	0	0
Blurred vision	2 (5.7)	0	0	0
Dizziness	2 (5.7)	0	1 (7.7)	0

ALT, alanine transaminase; AST, aspartate transaminase.

## Discussion

The present study retrospectively analyzed the treatment efficacy between crizotinib and ceritinib in the front‐line setting in a directly comparative manner. Patients who received ceritinib were associated with a significantly longer PFS than those who received crizotinib. Analysis of the pattern of disease progression between the two drugs showed that the higher treatment efficacy of ceritinib was mainly attributed to a significantly better control of systemic progression. Gastrointestinal symptoms and elevated liver function were the major adverse effects which accounted for the toxicity profiles of ceritinib and crizotinib treatment, respectively.

In the absence of a randomized head to head study comparing the efficacy between ceritinib and crizotinib in the first‐line setting, previous studies addressed this by taking advantage of an indirect comparative approach using the patient cohort propensity score‐matched from the ASCEND 4 and PROFILE 1014 trials.^4^, ^14^ In this indirect comparison, ceritinib showed a significantly better treatment efficacy than crizotinib.^19^, ^20^ Although this approach provided preliminary and insightful findings, it remained limited by the unadjustable bias inherent in each trial. The present study, otherwise analyzed in a manner of direct comparison, confirmed the finding noted in the previous indirect comparison.

An impressive efficacy of ceritinib with a PFS of 32.3 months was noted in the present study. This finding can be partly associated with the Asian ethnicity of our study population. A previous study of ceritinib treatment in the first‐line setting, the ASCEND 4 trial, had reported a PFS of 16.6 months. However, when the patients of Asian heritage were analyzed separately, a longer PFS up to 26.3 months was noted.^14^ The influence of ethnicity on the therapeutic efficacy was not limited to the ceritinib treatment arm, but also to the control arm of chemotherapy in which the PFS of chemotherapy for Asian patients was 9.7 months compared to a PFS of 8.1 months for the overall population. On the other hand, the crizotinib treatment group of the present study also showed a longer PFS of 12.9 months compared to the PROFILE 1014 trial.^4^ Recently, the association between Asian ethnicity and efficacy of crizotinib treatment in the front‐line setting has also been reported. Nishio *et al*. reported a PFS of 13.6 months and a 56% hazard reduction of disease progression and death for Asian patients in the PROFILE 1014 compared to a PFS of 9.6 months and a 48% hazard reduction for non‐Asian patients in the same study.^24^ Taken together, the present study demonstrated a good efficacy of ceritinib and crizotinib treatment for Asian Taiwanese patients with ALK‐positive NSCLC, with a superior efficacy in favor of ceritinib treatment.

The better therapeutic efficacy of ceritinib relative to crizotinib can be partly associated with a broader coverage of ceritinib treatment for multiple tumor clones, particularly for those with crizotinib‐resistant ALK mutations. This can be understood by considering a similar scenario of the epidermal growth factor receptor gene (*EGFR*) mutated NSCLC treated by osimertinib relative to gefitinib/erlotinib in the front‐line setting.^25^ Given osimertinib is a highly active agent for the treatment of gefitinib/erlotinib‐resistant T790M tumor clone, the front‐line administration of osimertinib allows the early eradication of T790M part within an *EGFR*‐mutated tumor and thereby significantly improves tumor control relative to the front‐line use of gefitinib/erlotinib. The successful suppression of the T790M tumor clone by the front‐line use of osimertinib can also be suggested by the subsequent mechanisms of acquired resistance in which the T790M‐mediated resistance can no longer be identified.^26^

In line with the above mentioned context, an in vitro study has shown that ceritinib was highly active against tumor clones L1196M, G1269A, C1156Y and I1171T/N/S, which were the top four crizotinib‐resistant mechanisms associated with the acquired ALK mutations.^27^, ^28^, ^29^ Therefore, the early eradication of these tumor clones by the front‐line administration of ceritinib may explain the better therapeutic efficacy, especially a favorable control of systemic progression, than the front‐line use of crizotinib. Furthermore, it is of note that when the crizotinib‐resistant tumor clones L1196M, G1269A, C1156Y and I1171T/N/S were treated with alectinib, they were not as sensitive as those treated by ceritinib in the in vitro study.^27^ Whether this could possibly be an explanation for the earlier finding of the ALEX trial in which the front‐line use of alectinib, when compared to crizotinib, only showed an equivalent capacity for the control of systemic progression warrants further investigation.^10^

Although the present study had its inherent limitations of being of a retrospective nature with a small sample size, it is of value given the lack of a direct head to head comparison between ceritinib and crizotinib. In addition, this analysis also suggested that the differential therapeutic efficacy between the two drugs when they were administered in the front‐line to Asian patients should actually be massive, and therefore the difference could be easily discerned, even if the analysis only involved a small group of patients.

In conclusion, this study demonstrated that the administration of ceritinib in the front‐line for the treatment of ALK‐positive NSCLC had superior efficacy than crizotinib, in which a better control of systemic progression could be attributed to this finding.

## Disclosure

No authors report any conflict of interest.
